# Sublethal effects of a rapidly spreading native alga on a key herbivore

**DOI:** 10.1002/ece3.8005

**Published:** 2021-08-16

**Authors:** Daniel J. Bradley, Jordi Boada, William Gladstone, Timothy M. Glasby, Paul E. Gribben

**Affiliations:** ^1^ School of Life Sciences Faculty of Science University of Technology Sydney Sydney NSW Australia; ^2^ Centre for Marine Science and Innovation Biological, Earth and Environmental Sciences University of New South Wales (UNSW) Kensington NSW Australia; ^3^ Institute of Aquatic Ecology Faculty of Sciences University of Girona Girona Spain; ^4^ NSW Department of Primary Industries Port Stephens Fisheries Institute Taylors Beach NSW Australia; ^5^ Sydney Institute of Marine Science Mosman NSW Australia

## Abstract

1. Multiple anthropogenic stressors are causing a global decline in foundation species, including macrophytes, often resulting in the expansion of functionally different, more stressor‐tolerant macrophytes. Previously subdominant species may experience further positive demographic feedback if they are exposed to weaker plant–herbivore interactions, possibly via decreased palatability or being structurally different from the species they are replacing. However, the consequences of the spread of opportunistic macrophytes for the local distribution and life history of herbivores are unknown.
2. The green alga, *Caulerpa filiformis*, previously a subdominant macrophyte on low intertidal‐shallow subtidal rock shores, is becoming locally more abundant and has spread into warmer waters across the coast of New South Wales, Australia.
3. In this study, we measured (a) the distribution and abundance of a key consumer, the sea urchin *Heliocidaris erythrogramma,* across a seascape at sites where *C. filiformis* has become dominant, (b) performed behavioral field experiments to test the role of habitat selection in determining the local distribution of *H. erythrogramma,* and (c) consumer experiments to test differential palatability between previously dominant higher quality species like *Ecklonia radiata* and *Sargassum* sp. and *C. filiformis* and the physiological consequences of consuming it.
4. At all sites, urchin densities were positively correlated with distance away from *C. filiformis* beds, and they actively moved away from beds. Feeding experiments showed that, while urchins consumed *C. filiformis,* sometimes in equal amounts to higher quality algae, there were strong sublethal consequences associated with *C. filiformis* consumption, mainly on reproductive potential (gonad size). Specifically, the gonad size of urchins that fed on *C. filiformis* was equivalent to that in starved urchins. There was also a tendency for urchin mortality to be greater when fed *C. filiformis*.
5. Overall, strong negative effects on herbivore life‐history traits and potentially their survivorship may establish further positive feedback on *C. filiformis* abundance that contributes to its spread and may mediate shifts from top‐down to bottom‐up control at locations where *C. filiformis* has become dominant.

## Graphical abstract

The spread of the previously subdominant *Caulerpa filiformis* along the coast of NSW, Australia has strong lethal and sublethal effects on a key herbivore population. We performed sea urchin surveys, behavioral experiments, and consumer experiments and found that *C* *aulerpa* has strong negative effects on their populations. These results may establish reinforcing feedbacks on the spreading algae and mediate shifts from top‐down to bottom‐up control.

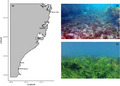

## 1 INTRODUCTION

In providing food and physical structure, macrophytes support biodiverse communities and complex food webs (Ellison et al., 2005; Jones et al., 1996; Lloyd et al., 2020). Globally, habitat‐forming macrophytes are threatened by a variety of abiotic and biotic disturbances, triggering transformations in the habitats they create (Dijkstra et al., 2019; Filbee‐Dexter & Wernberg, 2018; Krumhansl et al., 2016). Warming and heatwaves (thermal stress), changes in the nutrient regimes (i.e., eutrophication), and overgrazing by herbivores can all influence the distribution and abundance of habitat‐forming species (Cebrian et al., 2014; Filbee‐Dexter & Scheibling, 2014; Steneck et al., 2002, 2017; Wernberg et al., 2016). The loss of habitat‐forming macrophytes can result in the transition to alternate stable states in which communities are dominated by previously subdominant or novel species that may be more tolerant to disturbance. Moreover, the shift in macrophyte composition of the new community can result in changes to the strength of biotic interactions, for example, they may experience reduced rates of herbivory, compared with the species they replace, leading to further positive feedbacks even if the disturbance is removed (i.e., the passenger‐driver model:Bulleri et al., 2010; Feng et al., 2009; MacDougall & Turkington, 2005). Traditionally, changes in the strength of biotic interactions have focused on the implications for the abundance or the distribution of macrophytes (Boada et al., 2017; Pessarrodona et al., 2019) and much less is known about the consequences for the herbivores, although strong negative effects on herbivores may be predicted if the novel macrophytes are more resistant to herbivory (but see Boudouresque et al., 1996; Felline et al., 2012).

Both the physical and biological properties of macrophytes can mediate changes in the distributions of herbivores via lethal or sublethal effects (e.g., behavioral or life‐history traits, Lubchenco & Gaines, 1981). If one macrophyte is replaced with another that is structurally and biologically similar, then consumer‐mediated interactions may not change dramatically (Buschbaum et al., 2006; Steneck & Dethier, 1994). If, however, the new habitat is structurally different, this may have large impacts on associated species. For example, scouring or whiplash by algae can limit the access of grazers to the substratum (Konar & Estes, 2003). The growth form of vegetation can affect the abundance and movement of herbivores (Bach, 1981; Lanham et al., 2015; McGuinness & Underwood, 1986) while the quality of vegetation may also influence herbivore behavior (Livore & Connell, 2012b; McArthur et al., 2014; Weterings et al., 2018). Herbivores may avoid a less palatable species or those with lower nutritional quality, thereby affecting their distribution and abundance across land and seascapes (Kriegisch et al., 2019). In cases where the less palatable food source is a novel spatially dominant species, consumers may have no option but to consume the less preferred species with sublethal effects (e.g., reduced growth, biomass, or reproductive investment, Gribben & Wright, 2006; Tomas et al., 2011; Wright et al., 2011). Separate studies have shown that the invasive macrophytes can have reduced palatability compared with native macrophytes, which can release the macrophytes from predation pressure (e.g., Cacabelos et al., 2010; Nejrup et al., 2012), but also alter the distribution, behavior, and performance of native herbivores (see Scheibling & Anthony, 2001; Tomas et al., 2011). In other cases, however, native urchin grazers did consume introduced algae at greater or similar rates to native algae (e.g., Cacabelos et al., 2010; Noè et al., 2018). More recently documented is the spread of native macrophytes in response to environmental changes, some of which are having ecological effects that are analogous to those of invasive species. If invasive macrophytes have similar palatabilty to native macrophytes, we predict that the consequences for herbivores in the invaded region will be negligible. Globally, however, the consequences for herbivores in regions where native‐invaders (sensu Simberloff, 2011) are colonizing novel regions are unknown.

On the east coast of Australia, the top‐down effects of sea urchin grazing create and maintain extensive barrens (devoid of macroalgae). Barrens are typically created by the large diadematid urchin *Centrostephanus rodgersii,* which grazes on brown algae (Andrew & Byrne, 2007; Fletcher, 1987; Hill et al., 2003). Smaller patches of barrens can also be created by large densities of the echinometrid urchin *Heliocidaris erythrogramma*, one of the most common herbivores along the Great Southern Reef (Bennett et al., 2016; Keesing, 2020) particularly in shallower, more sheltered waters (Connell & Irving, 2008; Ling et al., 2019; Wright et al., 2005). In smaller densities, *H. erythrogramma* may not create barrens but can still remove canopy‐forming algae such as *Ecklonia radiata, Sargassum* spp., and *Cystophora* spp. (Livore & Connell, 2012a). Wright et al. (2005) observed the removal of the articulated coralline *Amphiroa anceps* and the fleshy brown *Zonaria diesingiana* by large numbers of *H*. *erythrogramma*, while in laboratory feeding trials, they found that the urchin also fed on *Corallina officinalis*, *S. vestitum,* and, to a lesser extent, the chemically defended rhodophyte *Delisea pulchra*. In Western Australia where *H. erythrogramma* seems to feed primarily on drift algae, the urchin is known to preferably retain *E. radiata* (Kriegisch et al., 2019; Vanderklift & Kendrick, 2005). On exposed rocky shores, *H. erythrogramma* generally stay close to or within crevices to avoid predation or dislodgement by waves. Where crevices are not available, *H. erythrogramma,* like other urchins, often bore into the rock effectively creating their own custom shelters (Russell et al., 2018). Urchins generally continue to bore as they grow, often ultimately creating a shelter in which the entrance is smaller than the urchin itself, confining them to their crevice. In this situation, urchins tend to feed on drift macroalgae (Keesing, 2020; Kriegisch et al., 2019).

The shallow rocky reef habitat of *H. erythorogramma* is becoming increasingly colonized by the native green alga *Caulerpa filiformis* in New South Wales, Australia. *C. filiformis* was previously known to be a subdominant community member and have a distribution restricted to about 300 km (Glasby et al., 2015). It has undergone a pronounced expansion and is now becoming more conspicuous within its range (Glasby, et al., 2015; Voerman et al., 2017) and having spread 500 km outside its known distribution. Unusually, it is spreading northwards into warmer waters (Glasby et al., 2015). At many sites, it forms large dense, monospecific stands and may be the most dominant macrophyte present. Beds of *C. filiformis* trap sediment (Voerman et al., 2017; Zhang et al., 2014), which occupies the rocky surface and fills in bore holes normally utilized by herbivores. Observations and small‐scale experiments suggest that on many rocky reefs, *C. filiformis* is replacing turf‐forming coralline algae and species of *Sargassum* (Voerman et al., 2021; Zhang et al., 2014). *C. filiformis* is structurally different from the native algae that it is apparently replacing, having much longer fronds than coralline turf, highly chemically defended, and more compact than species of *Sargassum* spp. As such, the spread of *C. filiformis* may affect the behavior or life history of *H. erythrogramma*. In this study, we combined a variety of disparate approaches (feeding preference assays, field‐behavior experiments, and laboratory life‐history experiments) to determine the effect of *C. filiformis* on the local distribution and abundance of *H. erythrogramma*.

We used large‐scale surveys to determine spatial patterns in abundance of *H. erythrogramma* at sites dominated by *C. filiformis*, and field (movement) experiments and laboratory (feeding trials) to determine if abundance patterns could be related to any lethal or sublethal effects of *C. filiformis* on urchins. We tested the hypotheses that because *C. filiformis* is chemically defended and structurally different from algae it replaces (a) *H. erythrogramma* abundance and homing scar occupancy would decline from outside to inside patches of *C. filiformis*, (b) *H. erythrogramma* not confined to homing scars would actively avoid beds of *C. filiformis*, (c) *H. erythrogramma* would preferentially consume other native macrophytes over *C. filiformis,* and (d) consumption of *C. filiformis* would have lethal and/or sublethal effects on *H. erythrogramma*.

## 2 METHODS

### 2.1 Species and study locations

*Caulerpa filiformis* is a coenocytic green alga found on intertidal and subtidal reefs in Australia, Peru, and South Africa (Glasby, et al., 2015). In New South Wales, Australia, *C. filiformis* occurs between 0 and 6 m water depth, primarily on rocky substrata in exposed and sheltered locations (e.g., inside Sydney Harbour) where it retains a thick layer of sediment (Voerman et al., 2017, 2019). Its root‐like rhizomes form dense, entangling mats, which can trap sediment (Voerman et al., 2017), and the flattened blades grow to >40 cm long with high variation in morphology in relation to local settings (Voerman et al., 2019).

**FIGURE 1:**
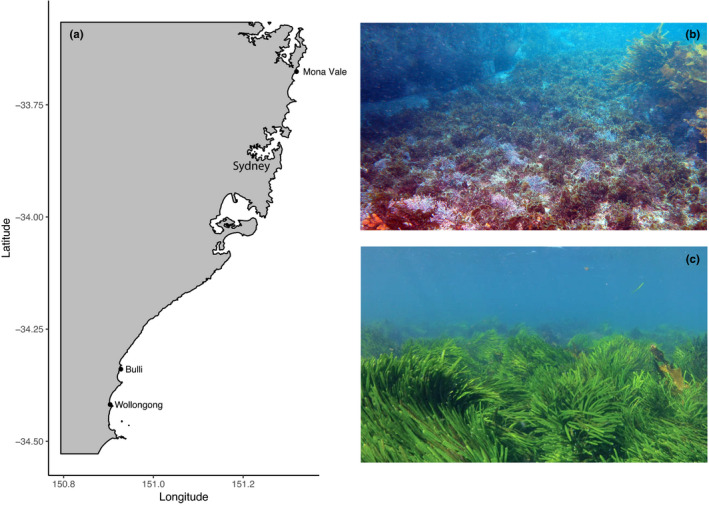
Map of study locations (i.e., Mona Vale, Bulli, and Wollongong) on the east coast of Australia (a), New South Wales. The top right photograph shows a shallow rocky landscape dominated by turfing algae with the presence of *Sargassum* spp and *Eklonia radiata* (b) while the bottom right photograph (c) shows Mona Vale, dominated by *C. filiformis*

Surveys and field experiments in this study were conducted at three locations (i.e., Mona Vale, 33°40′33″S, 151°19′08″E; Bulli, 34°20′21″S, 150°55′39″E; and Wollongong, 34°25′05″S, 150°54′13″E, see Figure 1a) covering approximately 150 km along the temperate coast of New South Wales (NSW), Australia. All locations were open coastline with extensive reefs extending from the shallow intertidal to depths of ~5–10 m. Reefs contained varied substrata, including rocky areas with boulders and flat sections, vegetated areas consisting of large monospecific beds of *C. filiformis* (as in Figure 1c) and sections of mixed turf‐forming algae consistently of different species with dominance by geniculate corallines that trap sediment (Connell et al., 2014), and the presence of *Sargassum* spp. and *Ecklonia radiata* (as in Figure 1b).

### 2.2 Patterns of urchin abundance inside and outside *C. filiformis*

At each of the three locations, in water between 0 and 2 m, we compared the abundance of *H. erythrogramma* and the number of homing scars on rocky platforms within patches of *C. filiformis* to those in surrounding algal habitats (“outside”), which consisted primarily of geniculate coralline algae and sparsely distributed browns such as *Sargassum* spp. and *E. radiata*. Patches of *C. filiformis* were a minimum of ~3 m × 3 m and separated by at least 10 m. Within *C. filiformis* patches, urchins and the number of homing scars were also compared between two positions (“inside” and “edge”). Edges were defined as areas within 1 m of the border of a patch of *C. filiformis*, while “inside” areas were in central section of patches, >1.5 m from the border. All the urchins found were sitting in self‐bored homing scars or rock crevices. Mainly one large monospecific stand of *C. filiformis* dominates the locations with presence of secondary smaller patches. Abundances of *H. erythrogramma* and homing scars were counted in *n* = 5 replicate quadrats (*N* = 1 per patch; 50 × 50 cm) in each position with respect to patches (inside, edge and outside) at each location. Quadrats were haphazardly placed within patch positions. Quadrats were carefully searched for urchins and homing scars, which involved removing dense vegetation and, in some cases, feeling through sediment among *C. filiformis* to count the number of homing scars. It is possible that some homing scars were missed among dense *C. filiformis*.

### 2.3 Urchin habitat preferences

To determine how *C. filiformis* influenced urchin movement, we collected ~80 *H. erythrogramma* of ~5 cm test diameter (TD, without spines) from a shallow rocky reef in Chowder Bay, Sydney Harbour (33°50′24.5″S 151°15′16.1″E). In order to relocate and identify experimental urchins, we tagged them. To do this, we used a hypodermic needle (14 G) to pierce urchins through the test and placed a fishing line through it. The needle was then removed, and the line tied off. This technique has been extensively used to tag sea urchins and does not affect their survival (Boada et al., 2015). On the other end of the fishing line, we attached numbered plastic tags to identify an individuals' starting point in the experiment (i.e., inside, edge, or outside *C. filiformis*). We placed *H. erythrogramma* in tanks of free‐flowing sea water after tagging and left them to recover for 48 hr. No urchins died as a result of this process. Urchins were then placed into the field at the Mona Vale location. We haphazardly placed 20 urchin individuals in the middle of a large *C. filiformis* patch (~500 m^2^, approximate size estimated in situ by divers), 20 on the edge, and 20 outside, among primarily coralline and sparsely distributed browns such as *Sargassum* spp. For this experiment, the inside position was 1 m from the edge. Urchins on the edge were placed immediately adjacent to *C. filiformis*, in the interface between the patch and the neighboring habitat and urchins outside were placed at ~1 m from the edge. All urchins were randomly allocated to positions within the patch at sufficient distance (at least 2–3 m apart) to maintain independence among individual urchins. One hour after placing urchins, we recorded the position of all tagged urchins as either middle, outside, or on the edge of a *C. filiformis* patch. The urchins moved very quickly once placed in the field (up to 0.5 m within the first 2 min) so 1 hr was adequate time to assess movement patterns.

### 2.4 Feeding preferences of *H. erythrogramma*

We conducted a series of mesocosm experiments at the Sydney Institute of Marine Science (SIMS) to test the feeding preference of *H. erythrogramma*. All the urchins used in the experiments were collected from Bare Island (33°59′36″S, 151°13′54″E), where *H. erythrogramma* occur in high densities (50–100 m^−2^, see Wright & Steinberg, 2001). We did not starve urchins prior to the start of the experiment. We first conducted no‐choice (i.e., a single food item) experiments and then a set of preference feeding assays with two species at a time. We used experimental containers (30 × 30 × 15 cm; ~4 L) supplied independently with filtered free‐flowing sea water (300 µm). For no‐choice (i.e., single food item) experiments, we placed one urchin (~5 cm TD) together with a ~5 g piece of either *C. filiformis*, *E. radiata,* or *Sargassum vestitum* in each container (*n* = 20). Algae were blotted dry to constant wet weight. We also visually assessed all algae to ensure they were free of epiphytes before being offered to urchins in containers. Experimental controls (*n* = 10 per treatment) to test for natural degradation of algae were created by placing five grams of each seaweed in containers without *H. erythrogramma*. After 72 hr, we removed any remaining seaweed from containers, blotted dry to constant wet weight, and recorded the final weights. We used the same methods for preference (choice) feeding assays except we crossed the different algae into pairwise treatments (*C. filiformis* × *E*. *radiata, C. filiformis* × *S*. *vestitum*. and *E. radiata* × *Sargassum* spp. *n* = 20 pairs/experiment). When presented with two species, *H. erythrogramma* were placed in the center of the container with algal species at either end. Water flow in the containers was directed as such to cause minimal interference to algae upon initiation of the experiments. After 48 hr, any remaining algae were collected and reweighed as above.

### 2.5 Effects of *C. filiformis* on urchins

To test the effects of *C. filiformis* consumption on urchins, we performed an additional experiment at SIMS. We placed 30 individuals of *H. erythrogramma* ~5 cm test diameter in each of nine 54 L tanks with free flowing filtered 300 µm seawater, which reflected urchin densities observed in the studied region 20–80 ind./m^2^, Wright & Steinberg, 2001). We randomly assigned treatments to each of the tanks in which urchins were fed with either nominally low‐quality food (*C. filiformis*), high‐quality food (*E. radiata*), or nothing (starvation; *n* = 3 tanks per treatment). Algae were supplied to tanks ad libitum over the course of the experiment and replaced/replenished seaweed, and removed older uneaten algae when necessary for a total of 18 weeks. During the experiment, we measured urchin mortality rates and, at the conclusion of the experiment, analyzed total mortality, total weight, gonad weight, and test weight of surviving urchins. Of the urchins that survived, we sacrificed a total of 67 urchins (25 fed with *E. radiata*, 35 fed with *C. filiformis,* and 7 starved) and separated calcareous material from gonads. A blockage in a water line resulted in the loss of all individuals in one of the *E. radiata* tubs resulting in an unbalanced number of urchins from different treatments. Gonads and calcareous material were placed in preweighed weigh trays and dried at 60℃ for 48 hr before being reweighed.

### 2.6 Statistical analysis

We analyzed urchin abundance, number of homing scars, and percent homing scar occupancy using generalized liner mixed models with position in the seascape (middle, edge, and outside *C. filiformis* patches) as a fixed factor and location as a random factor. All models were fit (random intercepts) to a Poisson distribution. Similarly, we used a generalized linear model with a Poisson distribution to test the differences between the numbers of urchins positioned in different habitats at the end of the movement experiment. For no‐choice experiments, we used linear models to test for differences in algal consumption. In two‐choice experiments, we used Wilcoxon tests to identify preferences between the different species offered. To evaluate mortality, we used a GLM with a binomial distribution (urchins dead or alive at the end of the experiment) considering food item as fixed factor and the aquaria as random factor. Finally, to evaluate the physiological consequences of eating *C. filiformis* compared with other seaweed or nothing, we used a set of linear mixed models to evaluate the effect of food source on the calcareous weight, gonad weight and the ratio (as a proportion) of gonad weight and calcareous weight. In all the cases, we set the seaweed used to feed urchins as a fixed factor and the tub in which urchins were placed was set as a random factor. We applied a log +0.01 transformation to values of calcareous weight, gonad weight, and the ratio between them. We used Tukey's test to explore pairwise differences when required. We used the open source R software from R‐CRAN and packages lme4 and ggplot2 to perform all the statistical analyses and plots (Bates et al., 2011; R Core Team, 2020).

## 3 RESULTS

### 3.1 Patterns of urchin abundance inside and outside *C. filiformis*

**FIGURE 2:**
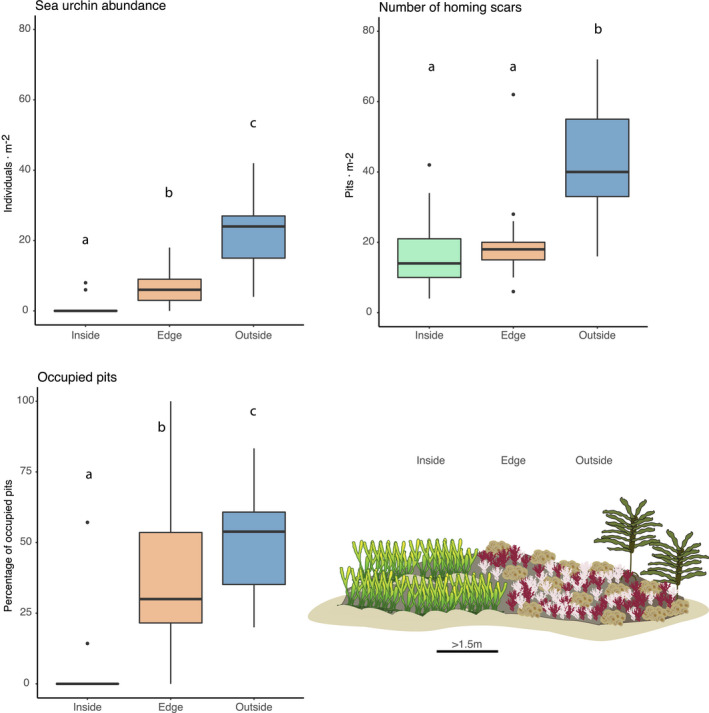
(a) Density of urchin *H. erythrogramma* in the studied region inside, at the edge and outside *C. filiformis* patches. (b) Density of homing scars inside, at the edge and outside *C. filiformis* patches. (c) Percentage of homing scars occupied by an individual of *H. erythrogramma*. Data in plots represent the values for all 3 locations

The abundance of *H*. *erythrogramma* differed among all three habitats and was highest outside (22.13 ind/m^2^ ± 2.61 *SE*) patches of *C. filiformis,* followed by patch edges (7.2 ind/m^2^ ± 1.44 *SE*) and almost completely absent inside (0.93 ind/m^2^ ± 0.64 *SE*) patches (Figure 2a, *p* < .01). The total number of homing scars recorded was highest outside (44.4 homing scars/m^2^ ± 4.29 *SE*) *C. filiformis* but did not differ between the middle (17.87 homing scars/m^2^ ± 3.19 *SE*) and edge (20 homing scars/m^2^ ± 3.32 *SE*) of patches (Figure 2b, *p* < .01). However, the percentage of homing scars occupied by live urchins was lower in the center of *C. filiformis* compared with the edges or outside patches, which did not differ from each other (Figure 2c, *p* < .01). Patterns were consistent across locations.

#### 3.1.1 Effects of *C. filiformis* on urchin movement

**FIGURE 3:**
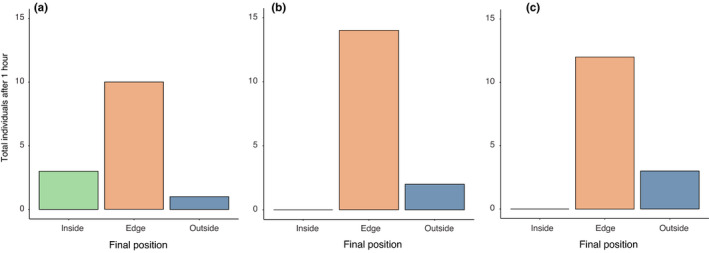
Number of urchins found at each position at the end of experiment of those place (a) inside *C. filiformis*, (b) at the edge between the two habitats, and (c) outside *C. filiformis* patches at the beginning of the experiment

After 1 hr, 45 of the 60 tagged urchins were found and there were significant differences in the number found in each position (*p* < .01). Contrary to our predictions, we found only 10% of the urchins outside *C. filiformis*. Regardless of the position urchins were originally placed in, most of them moved to the edge between *C. filiformis* and the adjacent habitats of coralline and sparsely distributed brown algae (Figure 3). Of all the urchins, 60% ended up at the edge of the two habitats. As predicted, few (5% of all urchins) remained within patches of *C. filiformis* at the end of the experiment and none of these had moved from either the edge or outside.

#### 3.1.2 Effects of *C. filiformis* on urchin feeding preference and performance

**FIGURE 4:**
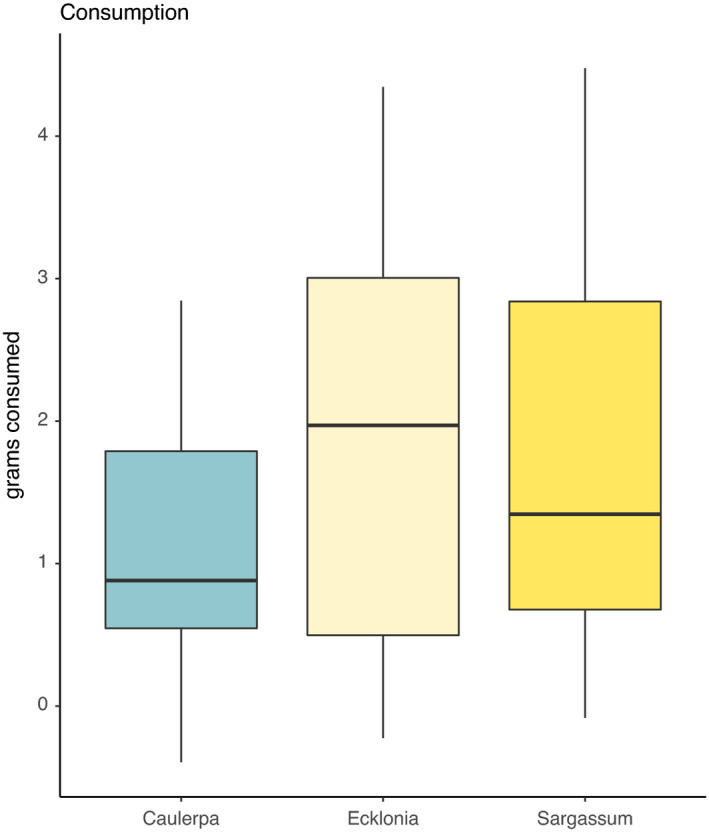
Urchin consumption (grams consumed) of each of *C. filiformis*, *E. radiata* and *Sargassum* spp in no‐choice food assays. Dark lines in boxplot represent the median values found per treatment

The amounts of *C. filiformis*, *E. radiata,* and *Sargassum* spp. consumed by *H. erythrogramma* did not differ in no‐choice feeding trials (Figure 4, *p* > .05), although there was a tendency for reduced consumption of *C. filiformis* (compared with other algae, Figure 4).

**FIGURE 5:**
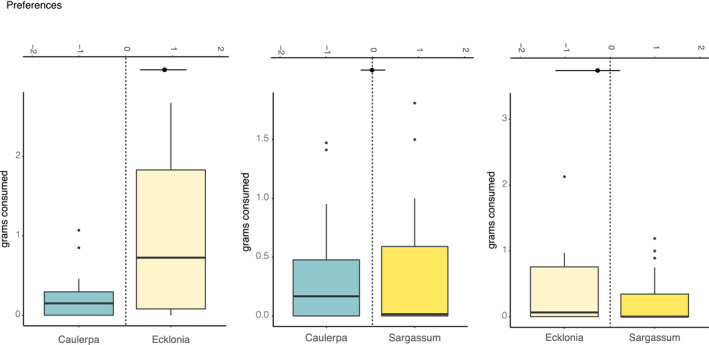
Two‐choice experiment results when urchins were offered (a) *E. radiata* & *Sargassum* spp, (b) *C. filiformis* & *Sargassum* spp, and (c) *C. filiformis* & *E. radiata* in total grams consumed. The black line in boxplots represents the median values. The top *x*‐axis represents the Wilcoxon test results on the preferences between the two‐choice items

Across all preference trials, *H. erythrogramma* consumed approximately four times as much *E. radiata* as either *C*. *filiformis* or *Sargassum* spp. When presented with a choice, urchins consumed significantly more *E. radiata* than *C. filiformis* (Figure 5). There was, however, no difference in consumption of *C. filiformis* and *Sargassum* spp. Similarly, urchin consumption of *Sargasssum* spp. and *E. radiata* did not differ, although there was a tendency for urchins to consume more *E. radiata* (Figure 5).

#### 3.1.3 Lethal and sublethal effects of *C. filiformis* on urchins

**FIGURE 6:**
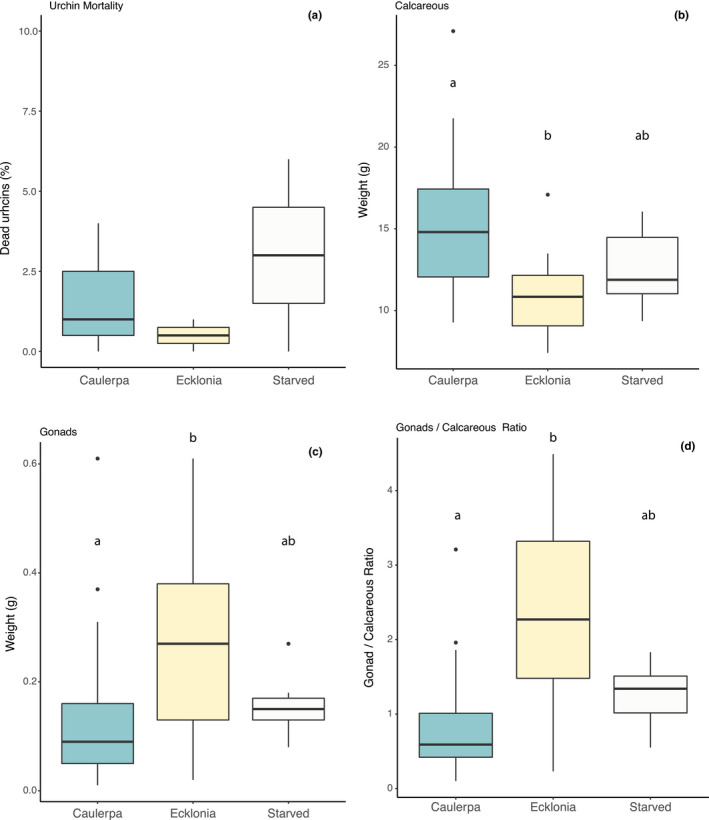
Lethal and nonlethal effects of feeding on *C. filiformis* compared with *E. radiata* or starved. (a) Sea urchin mortality at the end of the experiment, (b) calcareous body proportion weight in grams, (c) gonad weight in grams, and (d) the ratio of gonad to calcareous body weight

Although results were not statistically significant, probably due to missing replicates for the *E. radiata* treatment, mortality was higher for starved urchins (10% ± 5.77 *SE*) and urchins fed with *C. filiformis* (5.56% ± 4.01 *SE*) compared with those fed with *E. radiata* (1.67% ± 1.36 *SE*) (Figure 6a). Of those urchins that survived, dried calcareous weight was higher for urchins fed with *C. filiformis* (15.17 g ± 0.66 *SE*) compared with *E. radiata* (10.82 g ± 0.44 *SE*) but did not differ between urchins fed *C. filiformis* or starved (12.61 g ± 0.91 *SE*), or starved and *E. radiata* fed urchins (Figure 6b, *p* < .01). However, the ratio of gonad to calcareous weight was lower for urchins fed *C. filiformis* (0.13 g ± 0.02 *SE* dried weight) compared with *E. radiata* (0.27 g ± 0.03 *SE*), although there were no differences in either of these measures between the algae fed and starved urchins (0.15 g ± 0.02 *SE*, Figure 6c).

## 4 DISCUSSION

Globally, many marine foundation species, including macrophytes, are under increasing stress caused by main diverse set of disturbances including herbivory (Filbee‐Dexter & Scheibling, 2014; Krumhansl et al., 2016; O'Brien & Schiebling, 2016). These habitats are commonly being replaced by opportunistic, subdominant species of turfing algae. On the east coast of Australia, conversion to turfing habitat may be important for aiding the spread and increase in local abundance of *C. filiformis* (Voerman et al., 2021). Here, we show that the sea urchin *H. erythrogramma*, an abundant herbivore in the region, actively avoids dense areas of this expanding habitat and that prolonged consumption of *C. filiformis* results in reallocation of energy, reduced gonad size, and a tendency for increased mortality. Although gonad condition is known to relate to food quality in *H. erythrogramma* and other urchins (Andrew, 1986; Andrew et al., 2007; Livore & Connell, 2012b), we found little difference between effects of starvation and a diet of *C. filiformis*. Thus, *C. filiformis* likely experiences positive reinforcing demographic feedbacks from reduced herbivory, facilitating its stability and spread.

The physical properties of habitat‐forming species can have strong effects on associated organisms (Dijkstra et al., 2017; Gribben et al., 2020; Jones et al., 1996; Uyà et al., 2020; Wright & Gribben, 2017). Hori (2006), for instance, found that dense surfgrass beds (which are similar in structure to *C. filiformis* patches) were able to confine sea urchins to rock pools by limiting their movement. Specifically, vegetation can influence the foraging behavior of organisms living in the habitat they generate or alter the abiotic conditions of the environment (Bach, 1981; Underwood & Jernakoff, 1981). In our study, increasing abundances of urchins away from the center of *C. filiformis* patches appeared to be, at least in part, determined by the responses of urchins to *C. filiformis*. In our movement experiment, we found no urchins actively entering into *C. filiformis* patches. Instead, urchins concentrated at the edge between the two habitats possibly because the edge offers a good trade‐off between preferred food intake and structural protection against predators, although longer experiments will add further insights in this respect (see Farina et al., 2014). The fast movement rates contrast with previous literature suggesting the limited movement of this species (see Keesing, 2020). Thus, even the dense *C. filiformis* beds did not act as physical barriers to movement. Instead, urchins may avoid remaining within patches because homing scars are filled in by either rhizomes or sediment trapped by *C. filiformis* (Voerman et al., 2017). Indeed, most homing scars in *C. filiformis* were filled with sediment and were unoccupied by urchins. This supports Voerman et al. (2017) who suggested that *C. filiformis* may have overgrown areas of turfing algae, which were previously home to *H. erythrogramma*. In addition, reduced access to homing scars and increased sedimentation may force urchins to be located higher off the surface, making them more prone to predators or dislodgement via wave action (Pagès et al., 2013; Walker, 2007).

In addition to the physical effects, the presence of *C. filiformis* seems to also shape the distribution and abundance of urchins across the seascape through behavioral and physiological responses. *H. erythrogramma* is able to switch feeding modes in relation to food quality (from drift to grazing, Livore & Connell, 2012a). Similarly, the decrease in high‐quality food resources across a seascape and its substitution by less preferred species would force urchins to move to neighboring areas foraging for preferred food items. In this case, the preference for the more appealing *Ecklonia radiata* would stimulate these herbivores to move away from patches of *C. filiformis*, increasing pressure on the adjacent seaweed forest and indirectly enhancing the success of the spread of *C. filiformis*. However, when urchins remain confined to homing scars (Russell et al., 2018) or much of the reef is colonized by *C. filiformis*, urchins may be forced to feed on this food resource, resulting in sublethal (e.g., reduced reproductive capacity) and potentially lethal effects due to obliged consumption of drifting *C. filiformis*. This set of lethal and sublethal effects, probably associated with the secondary compounds typical of species from the genus *Caulerpa,* generally affect the feeding of different types of herbivores, (Boudouresque et al., 1996; Davis et al., 2005; Felline et al., 2012; Gollan & Wright, 2006; Miranda et al., 2019). Indeed, similar patterns have previously been described in the context of invasive species including species of the genus *Caulerpa* (see Scheibling & Anthony, 2001; Tomas et al., 2011).

Finally, we found that urchins reallocate energy from reproduction (gonads) to structural traits (test mass), suggesting negative consequences to the population, possibly via algal toxicity, of *C. filiformis* consumption, at least when compared to *E. radiata*. These results align with previous investigations that found clear links between resource allocation of *H. erythrogramma* and the habitat it occupies, and a clear hierarchy of resource allocation prioritizing gut and lantern then test and spines and finally gonads (see Keesing, 2020). Constable (1990) also found gonad resorption in individuals with low food availability. In our case, this diversion of energy away from reproduction when on a diet of *C. filiformis* was even more severe than when urchins were starved, possibly because of the secondary compounds of *C. filiformis,* which stresses the need for incorporating quantification of the sublethal effects associated with shifts in diet in ecological studies. These effects do not appear exclusive to the studied species interaction and may be similar for other herbivores in the area according to previous results showing clearly reduced consumption on *C. filiformis* (see Miranda et al., 2019). Our results suggest macrophyte ecosystem shifts to dominance of new primary producers may have critical consequences for the ecosystems functioning (e.g., herbivory, productivity).

Disturbance to algal competitors in shallow subtidal communities can facilitate the establishment and spread of invasive and opportunistic native algae (Zhang et al., 2014), which, once established, can cause further environmental change that facilitates their spread. *C. filiformis* appears to conform to this “passenger‐driver” model (Bulleri et al., 2010; Didham et al., 2005). Indeed, disturbance to algal communities is a key process allowing the initial establishment and expansion of *C. filiformis*. Our study suggests that its physical structure and reduced palatability may then contribute to reinforcing demographic feedbacks for *C. filiformis*. If herbivores reliant on macroalgae generally avoid *C. filiformis* as a food source then its continued expansion and increase in local abundance could cause a critical shift in the ecosystem functioning to bottom‐up control, although herbivorous fish abundances do not appear to be strongly affected by the presence of *C. filiformis* (Bradley et al., 2018), possibly because of their higher mobility. A recent global focus has been on describing the massive impacts range‐spreading herbivores have on algal communities through strengthening top‐down processes (Vergés et al., 2014, 2016). Here, we show that the opposite can occur, although the overall consequences for urchin populations and ecosystem function remain to be further determined. The stability of subdominant macrophytes after disturbances seems globally very high with foundation macrophytes having limited capacity to recover their original extension unless active management actions are applied (i.e., restoration, Bulleri et al., 2020; Wood et al., 2019). Therefore, if these findings are the rule rather than the exception, we may expect further global declines in the abundance of foundation macrophytes and an important shift in the energy flow in these ecosystems.

## CONFLICT OF INTEREST

The authors declare that they have no conflict of interests.

## AUTHOR CONTRIBUTIONS

**Daniel J. Bradley:** Conceptualization (equal); data curation (equal); formal analysis (equal); methodology (equal); writing‐original draft (equal); writing‐review & editing (equal). **Jordi Boada:** Conceptualization (equal); data curation (equal); formal analysis (equal); methodology (equal); visualization (equal); Writing‐original draft (equal); writing‐review & editing (equal). **William Gladstone:** Conceptualization (equal); writing‐orginal draft (equal). **Timothy M. Glasby:** Conceptualization (equal); formal analysis (equal); writing‐original draft (equal); writing‐review & editing (equal). **Paul E. Gribben:** Conceptualization (equal); data curation (equal); formal analysis (equal); funding acquisition (equal); investigation (equal); methodology (equal); project administration (equal); supervision (equal); writing‐original draft (equal); writing‐review & editing (equal).

## Supporting information

Table S1‐S4

## Data Availability

The data used in this study are publicly available at the Dryad repository (https://doi.org/10.5061/dryad.gmsbcc2nr).
