# Supplementary material for: Sublethal effects of a rapidly spreading native alga on a key herbivore
Source: Ecol Evol. 2021 Aug 16;11(18):12605–16. doi: 10.1002/ece3.8005 (PMC8462141; doi:10.1002/ece3.8005)
Supplement: Supplementary file 1 — Table S1‐S4 [file ECE3-11-12605-s001.docx]

**Sublethal effects of a rapidly spreading native alga on a key herbivore.**

D. J. BRADLEY*‡·; J. BOADA‡^·; W. GLADSTONE*; T. M. GLASBY^#^ AND P. E. GRIBBEN‡Ψ

*School of Life Sciences, Faculty of Science, University of Technology Sydney, Australia.

‡Centre for Marine Science and Innovation, Biological, Earth and Environmental Sciences, University of New South Wales (UNSW), Kensington, Australia.

^Institute of Aquatic Ecology, Faculty of Sciences. University of Girona. Girona, Spain.

^#^NSW Department of Primary Industries, Port Stephens Fisheries Institute, Taylors Beach, Australia.

ΨSydney Institute of Marine Science, 19 Chowder Bay Road, Mosman, NSW 2088, Australia.

**·** These authors have contributed equally to the study

**Summary of statistical results**

*Table 1. Generalized linear model results of field surveys on urchin abundance, number of homing scars and the percentage occupied by sea urchins. Position (within, at the edge or outside* C. filiformis*) was considered a fixed factor in all the models and location was considered random. Significant values are given in bold and pairwise comparisons are also detailed.*

|  | **Factor** | **Chisq** | **Df** | **Pr(>Chisq)** | **Pairwise comparison** |
| --- | --- | --- | --- | --- | --- |
| **Urchin abundance** | Position | 216.31 | 2 | **<0.01** | Inside < Edge < Outside |
|  |  |  |  |  |  |
| **Homing scars** | Position | 223.92 | 2 | **<0.01** | Inside = Edge < Outside |
|  |  |  |  |  |  |
| **Percentage of occupied homing scars** | Position | 370.43 | 2 | **<0.01** | Inside < Edge = Outside |

*Table 2. Generalized linear model results on the sea urchin movement. The final position of urchins was considered a fixed factor. Significant values are given in bold and pairwise comparisons are also described.*

|  | **Factor** | **Chisq** | **Df** | **Pr(>Chisq)** | **Pairwise comparison** |
| --- | --- | --- | --- | --- | --- |
| **Number of urchins** | Position | 42.382 | 2 | **<0.01** | Inside = Outside < Edge |

*Table 3. Linear model results for the no-choice consumption experiments. Seaweed species (food item) was included as fixed factor and test diameter as random factor.*

|  | **Factor** | **Chi Sq** | **Df** | **Pr(>F)** |
| --- | --- | --- | --- | --- |
| **Number of urchins** | Seaweed | 4.1477 | 2 | 0.1257 |
|  |  |  |  |  |

*Table 4. Linear mixed models results on the non-lethal effects of consuming* C. filiformis *on the sea urchin* H. erythograma*. In all the models the treatment (type of feed) was introduced as a fixed factor while the tub in which urchins were placed was included as random factor. Significant model values are given in bold and pairwise comparisons are detailed.*

|  | **Factor** | **Chisq** | **Df** | **Pr(>Chisq)** | **Pairwise comparison** |
| --- | --- | --- | --- | --- | --- |
| **Gonad weight** | Treatment | 21.285 | 2 | **<0.01** | Caulerpa = Ecklonia = Starved |
|  |  |  |  |  |  |
| **Calcareous weight** | Treatment | 58.066 | 2 | **<0.01** | Caulerpa = Starved = Ecklonia; Caulerpa > Ecklonia |
|  |  |  |  |  |  |
|  |  | **Sum Sq** | **Df** | **Pr(>F)** |  |
| **G/C index** | Treatment | 20.490 | 2 | **<0.01** | Caulerpa < Ecklonia = Starved; Caulerpa = Starved |
